# “It feels like a thousand tons” ‐ Embodiment of AD dementia risk

**DOI:** 10.1002/alz70858_106809

**Published:** 2025-12-26

**Authors:** Constanze Hübner, Carolin Schwegler, Annika Baumeister, Michelle Gerards, Yahveth Cantero‐Fortiz, Federica Ribaldi, Julia Braun, Frank Jessen, Ayda Rostamzadeh, Mercè Boada, Giovanni B Frisoni, Björn Schmitz‐Luhn, Christiane Woopen

**Affiliations:** ^1^ Center for Life Ethics, University of Bonn, Bonn, Germany; ^2^ University of Cologne, Cologne, Germany; ^3^ Department of Psychiatry and Psychotherapy, Medical Faculty, University of Cologne, Cologne, Germany; ^4^ Ace Alzheimer Center Barcelona – Universitat Internacional de Catalunya, Barcelona, Spain; ^5^ Geneva Memory Center, Geneva University Hospitals and University of Geneva, Geneva, Switzerland; ^6^ Center for Life Ethics, University of Bonn, Bonn, Nrw, Germany; ^7^ German Center for Neurodegenerative Diseases (DZNE), Bonn, Germany; ^8^ Excellence Cluster on Cellular Stress Responses in Aging‐Associated Diseases (CECAD), University of Cologne, Cologne, Germany; ^9^ Department of Psychiatry, Medical Faculty, University of Cologne, Cologne, Germany; ^10^ Networking Research Center on Neurodegenerative Diseases (CIBERNED), Instituto de Salud Carlos III, Madrid, Spain; ^11^ Ace Alzheimer Center Barcelona – International University of Catalunya (UIC), Barcelona, Spain; ^12^ Laboratory of Neuroimaging of Aging (LANVIE), University of Geneva, Geneva, Switzerland; ^13^ Center for Life Ethics, University of Bonn, Bonn, NRW, Germany

## Abstract

**Background:**

The predictive turn in Alzheimer's disease dementia (ADD) is marked by increasing research and use of biomarkers to determine individual ADD risk. It may even become available for the general population. Undergoing predictive testing and learning about the individual ADD risk is ethically relevant e.g., for (mental) well‐being and self‐perception and poses new challenges for clinical practice. The qualitative part of the PreTAD‐project (Predictive Turn in Alzheimer's Disease: Ethical, Clinical, Linguistic, Legal Aspects) analyzes these ethical aspects to derive meaningful implications for handling ADD risk estimation in cognitively unimpaired individuals.

**Method:**

In our qualitative study, individuals a) who had no prior experience with ADD, b) with first‐degree relatives with ADD, and c) with subjective cognitive decline created body maps alongside narrative interviews. Body mapping is a qualitative visual method by which we aimed to capture the embodied experience of hypothetically learning about (a, b) or a subjectively perceived (c) ADD risk. The body maps and interviews were systematically analyzed and triangulated.

**Result:**

In total, *N* = 28 participants created body maps (a, *n* = 8; b, *n* = 10; c, *n* = 10). The body maps provided deep, multifaceted insights into the embodiment of ADD risk. The focus on bodily sensations acted as a direct trigger for most participants to subjectively engage in a (hypothetical) scenario of being confronted with ADD risk. Participants experienced ADD risk as both a cognitive and bodily phenomenon, individually localized in different regions of their bodies (e.g., the heart). Common sensations were related to fear and anxiety or immediately perceived symptoms of cognitive decline, but also hope and urge to act, indicating a reflexive process of various sensations associated with ADD risk. Participants frequently used metaphors, e.g., describing heaviness (“a thousand tons”), to articulate their bodily sensations.

**Conclusion:**

The body maps alongside the participants’ narrations provided a multifaceted understanding of how bodily sensations, self‐perception, and mental state are interwoven and can be influenced when individuals are confronted with ADD risk. The findings highlight the connection between bodily, cognitive and emotional experiences, which is relevant for person‐sensitive risk communication. Body maps can inform ethical guidance for handling ADD risk estimation in clinical practice.

